# Taming the exoscope: a one-year prospective laboratory training study

**DOI:** 10.1007/s00701-023-05664-w

**Published:** 2023-06-27

**Authors:** João M. Silva, Oriela Rustemi, Donika Ivova Vezirska, Mika Niemelä, Martin Lehecka, Ahmad Hafez

**Affiliations:** 1 Department of Neurosurgery, Helsinki University Hospital and University of Helsinki, P.O. Box 266, FI-00029 Helsinki, Finland; 2 Department of Neurosurgery, Centro Hospitalar Universitário do Porto, Porto, Portugal; 3 Department of Neurosurgery, San Bortolo Hospital, Viale Rodofi 37, 36100 Vicenza, Italy; 4 Medical University of Sofia, Sofia, Bulgaria; 5 Bridge Hospital, Haartmaninkatu 4, PO Box 320, 00029 HUS, Helsinki, Finland

**Keywords:** Dissection, Exoscope, Microsurgical skill, Taming, Training program

## Abstract

**Purpose:**

Digital 3D exoscopes have been recently introduced as an alternative to a surgical microscope in microneurosurgery. We designed a laboratory training program to facilitate and measure the transition from microscope to exoscope. Our aim was to observe the effect of a one-year active training on microsurgical skills with the exoscope by repeating a standardized test task at several time points during the training program.

**Methods:**

Two board-certified neurosurgeons with no previous exoscope experience performed the same test tasks in February, July, and November during a 12-month period. In between the test tasks, both participants worked with the exoscope in the laboratory and assisted during clinical surgeries on daily basis. Each of the test segments consisted of repeating the same task 10 times during one week. Altogether, 60 test tasks were performed, 30 each. The test task consisted of dissecting and harvesting the ulnar and radial arteries of the second segment of a chicken wing using an exoscope (Aesculap AEOS). Each dissection was recorded on video and analyzed by two independent evaluators. We measured the time required to complete the task as well as several metrics for evaluating the manual skills of the dissection and handling of the exoscope system.

**Result:**

There was a clear reduction in dissection time between the first and the last session, mean 34 min (SD 5.96) vs. 26 min (SD 8.69), respectively. At the end of the training, both neurosurgeons used the exoscope more efficiently utilizing more available options of the device. There was correlation between the dissection time and several of the factors we used for evaluating the work flow: staying in focus, zoom control, reduction of unnecessary movements or repetitive manual motions, manipulation technique of the vessel under dissection, handling of the instruments, and using them for multiple dissection purposes (stretching, cutting, and splitting).

**Conclusion:**

Continuous, dedicated long-term training program is effective for microsurgical skill development when switching from a microscope to an exoscope. With practice, the micromotor movements become more efficient and the use of microinstruments more versatile.

**Supplementary Information:**

The online version contains supplementary material available at 10.1007/s00701-023-05664-w.

## Introduction

The use of the 3D digital exoscopes in neurosurgery has expanded significantly over the last few years. Exoscopes are increasingly employed in neurosurgical departments, offering new educational possibilities but demanding new ways of training for neurosurgeons [9, 10]. There are several systems on the market with different technical, software, and hardware characteristics. Despite their differences, all have the same goal to allow high quality microneurosurgery.

Exoscopes are cognitively demanding and impose physical differences when compared to the microscope. Switching from a microscope to an exoscope is not an easy task. Time and practice are required to become fluent with the new type of intraoperative magnification device [4, 5, 11, 14, 16, 17]. The operating room is not the right place for the initial training on a new surgical device. Practice environment simulating the movements and techniques of real surgery is a much better option. The simulator enables trainees and novice exoscopic neurosurgeons to practice their skills in a nonclinical environment numerous times without the risk of harm. Moreover, it provides trainees a platform to assess their abilities and keep track of their progress over time. Despite the general appreciation of the need for laboratory training, there is only limited knowledge on how and what to practice to improve one’s microneurosurgical skills when working with a novel device such as an exoscope.

Our aim is to develop a dedicated laboratory training program to facilitate and measure the transition from an microscope to an exoscope. For this purpose, we designed a simulator training task for novice exoscope users that was used to measure the skill progression. Our aim was to evaluate the effect of active continuous training with exoscope on microsurgical skills by repeating a standardized test task at several time points during a one-year training program. Our hypothesis was that through dedicated training the quality of the microsurgical work becomes better and more efficient. In this article, we describe our experiences of how long-term dedicated practice affects development of micromotor skills in novice exoscope users.

## Material and methods

The exoscope training program was carried out by two novice exoscope users (JS, OR), one-year skull-base, and vascular fellows at our unit. Both were board certified neurosurgeons with no previous experience on the use of exoscope but with experience of 7 and 12 years in microneurosurgery using an operating microscope. The two participants were tutored in both the exoscope use and the microsurgical training by the senior authors (AH, ML, and MN).

### Test setup

As a model, we used the dissection of a chicken wing. The test task was selected so that it required multiple integrated movements of both hands as well as control of the exoscope with a foot pedal. The task consisted of dissecting and harvesting the ulnar and radial arteries of the second segment of a non-frozen chicken wing. The aim was to dissect and isolate the ulnar and radial arteries while keeping the surrounding muscle tissue intact. The dissection was performed with integrated multi-maneuver dissecting technique using forceps and microscissors which is routinely used in, e.g., Sylvian fissure dissection [3]. All the tasks were performed using a 3D digital exoscope (Aeos Digital Microscope; Aesculap AG, Am Aesculap-Platz, 78532 Tuttlingen, Germany).

**Fig. 1:**
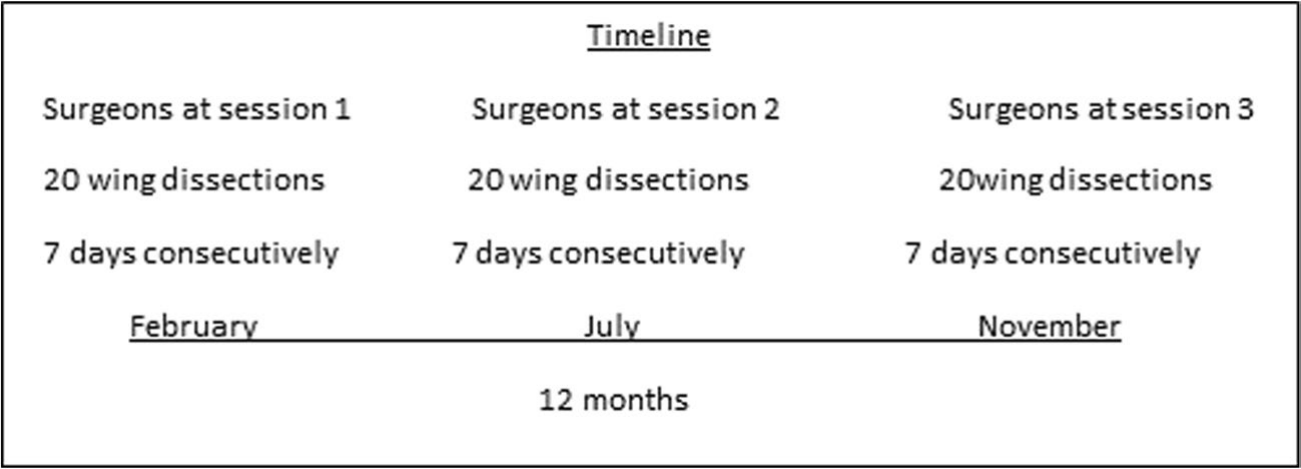
Timeline of experiment

First, the corresponding author (AH) demonstrated the dissection task to the participants (video 1). Afterwards, each participant repeated the task 10 times over the course of one year in three separate one-week segments in February, July, and November (Fig. 1). A total of 60 exoscope dissecting tasks were completed during this study. Each dissection was recorded on video for later evaluation.

In between the test tasks, both participants practiced on a daily basis both their microsurgical skills and the use of the exoscope. The practice consisted of dedicated laboratory practice on various models and assisting during complex skull-base and vascular procedures in the operating theater utilizing both surgical microscopes and exoscope.

### Video analysis

**Fig. 2:**
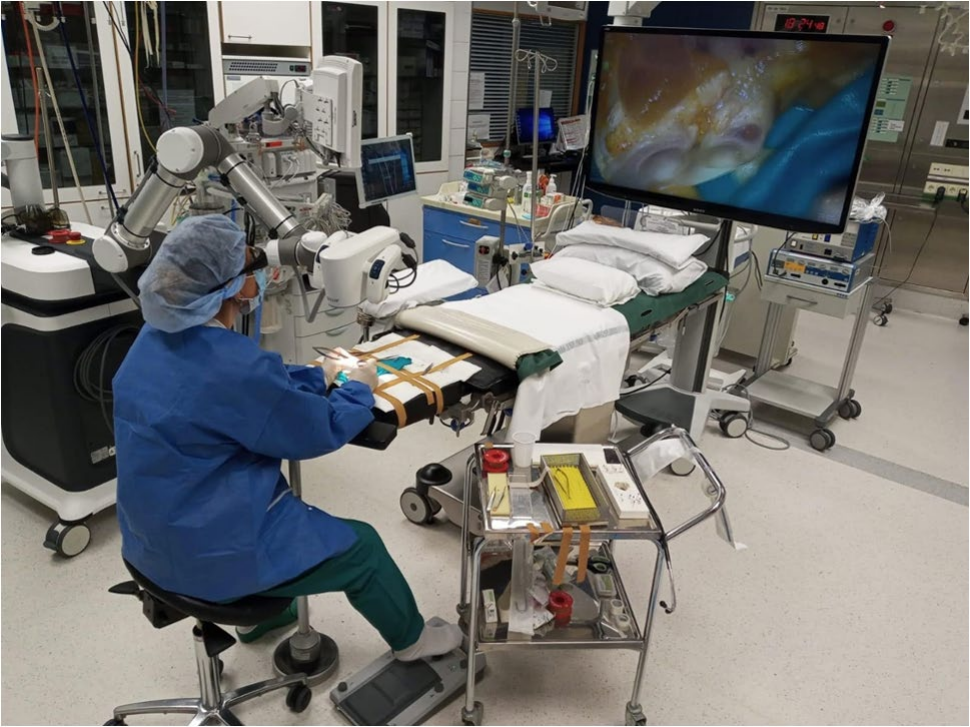
Overview of the preparation of the setup when using the three-dimensional exoscope. The surgeon operator is sitting looking horizontally at the three-dimensional wide-view monitor with 4K resolution placed in sight. The foot switch on the floor is used with microscope, in the operation room during the experimental dissection task. The working distance of the exoscope close to 250–300 mm

**Fig. 3:**
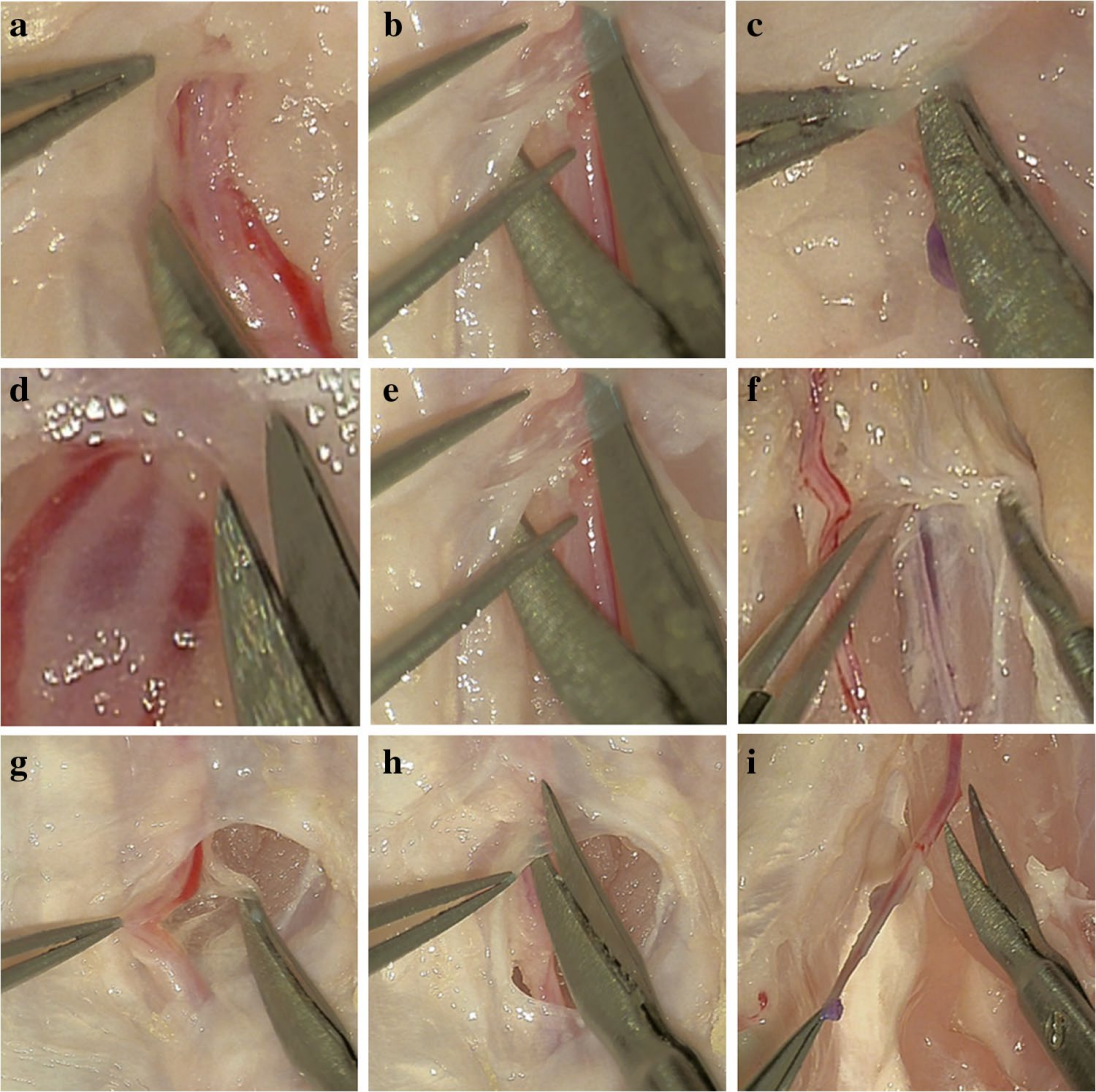
The dissecting techniques of harvesting ulnar and radial arteries from the second part of the chicken wing. **a** Using the scissors as retractor. **b** Using the scissors as dissector. **c** Using scissors as elevator. **d** Using scissors as paper knife. **e** Stretching the tissue. **f** Splitting. **g** Tearing. **h** Cutting. **i** Skeletonization of the artery

We evaluated the progress in two dimensions: (a) handling of the exoscope and the microinstruments and (b) refinement of fine motor skills under high magnification in a 3D video environment (Figs. 2 and 3a–i).

All the recordings were analyzed by two independent evaluators (DV and AH). The videos were analyzed in random order and blinded with respect to the surgeon. Initial agreement on the metrics of the evaluation was achieved by both reviewers analyzing several videos together in the beginning of the process.

The metrics used for video analysis are presented in Table 1.

**Table 1:** Task metrics

Metrics	Descriptions
Time to complete	From the first touch of an instrument until the arteries are isolated and cut out of the tissue [min]
Out of focus	Surgical field is out of focus [n]
Out of filed	Image is out of field [n]
Unnecessary movement	A movement which does not progress the work [n]
Zoom in	Increase in magnification [n]
Zoom out	Decrease of magnification [n]
Repeating	Movements repeated to reach the same goal [n]
Entry point	Attempts to find the entry zone of the dissected artery [n]
Skeletonize	Movements used to skeletonize the arteries [n]
Scissors as retractor	Microscissors used to retract tissues [n]
Scissors as dissector	Microscissors used to bluntly dissect tissue layers [n]
Scissors as elevator	Microscissors used to elevate tissue layers [n]
Scissor as paper knife	Micorscissors used to separate tissues like a paper knife [n]
Stretching	Stretching of tissue layers [n]
Splitting	Splitting of tissue layers [n]
Trabecular	Handling of the trabecular tissue around the vessels [n]
Forceps as spatula	Forceps was used to retract tissues [n]
Tearing	Separating of tissues by tearing [n]

*min* minute, *n* number of time

### Statistical analysis

The analysis focused on the changes in parameters over the training period. Data were pooled into three groups representing the three consecutive time periods during which the test tasks were performed. The metrics were reported as the means with standard deviations (SD).

The correlation between the time and the metrics in different sessions during the one year were assessed using the Pearson correlation coefficient. The *p*-values were calculated.

For the changes over time, the hypothesis test rejected the null hypotheses (0.001) while the significant level was 0.05. The asymptotic significances are displayed.

## Result

The results of the video analysis are presented in Table 2.

**Table 2:** Results of video analysis

	S1		S2		S3	
	Mean	SD	Mean	SD	Mean	SD
Time	34	5.96	33	7.86	26	8.69
Out of focus	18	8.69	16	13.85	11	10.44
Out of field	1	1.07	2	2.94	4	3.67
Unnecessary movement	9	7.08	13	6.95	11	7.17
Zoom in	8	4.33	5	2.30	3	1.83
Zoom out	5	2.80	3	1.83	2	1.46
Repeating	2	6.24	1	2.03	1	1.63
Entry point	13	4.33	10	2.36	7	2.44
Skeletonize	416	113.44	509	159.52	420	137.28
Scissors as retractor	2	2.00	1	3.19	1	2.08
Scissors as dissector	50	36.01	104	63.60	99	48.23
Scissors as elevator	12	12.65	9	15.06	8	9.78
Scissor as paper knife	24	18.35	32	32.71	39	51.62
Stretching	103	53.46	135	44.30	116	44.94
Splitting	87	34.29	198	166.29	162	148.03
Trabecular	15	7.68	19	14.64	14	12.97
Forceps as spatula	15	25.73	0	0.78	5	12.81
Tearing	2	1.94	1	0.75	1	1.41
Cutting	308	74.64	272	67.56	231	46.50

*S1* session 1 (February), *S2* session 2 (July), *S3* session 3 (November), *SD* standard deviation

The clearest finding was that dissection time showed continuous reduction over the course of the training period, mean 34 min vs. 33 min vs. 26 min for the three test segments, respectively.

**Fig. 4:**
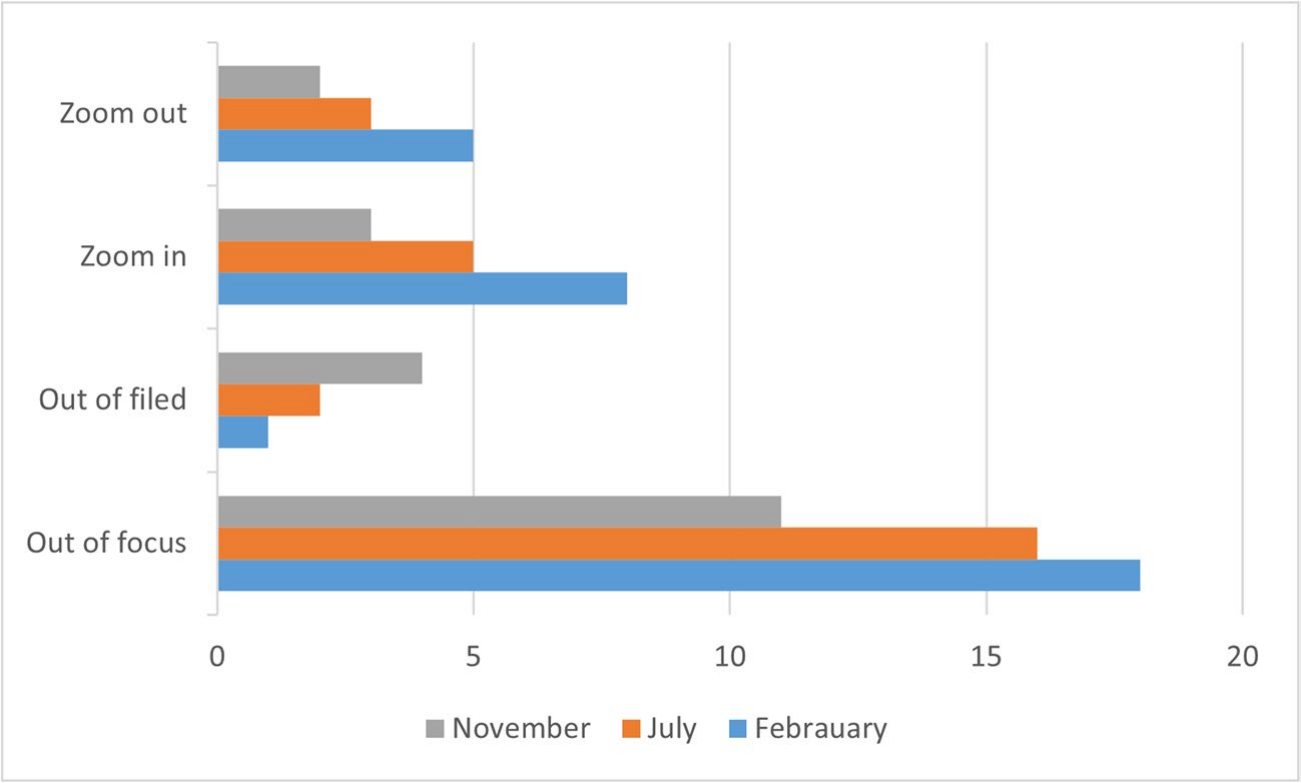
Metrics of exoscope use

We separately analyzed the metrics regarding the exoscope use, such as zoom out, zoom in, out of focus, and out of field (Fig. 4). Interestingly, the time shortened even though the exoscope was manipulated more often at the end of the training period. With increasing experience, the participants moved the exoscope more often around the surgical field, they used higher magnification, and they stayed more in focus. By the end of the experiment, most of the movements were performed using the foot pedal control. Zooming was used less frequently at the end of the trial as both participants stayed at higher magnification throughout the dissection instead of zooming in and out repeatedly. Higher magnification required more continuous movement of the exoscope which led to the exoscope being more often out of field for brief periods of time without increasing the overall dissection time.

**Fig. 5:**
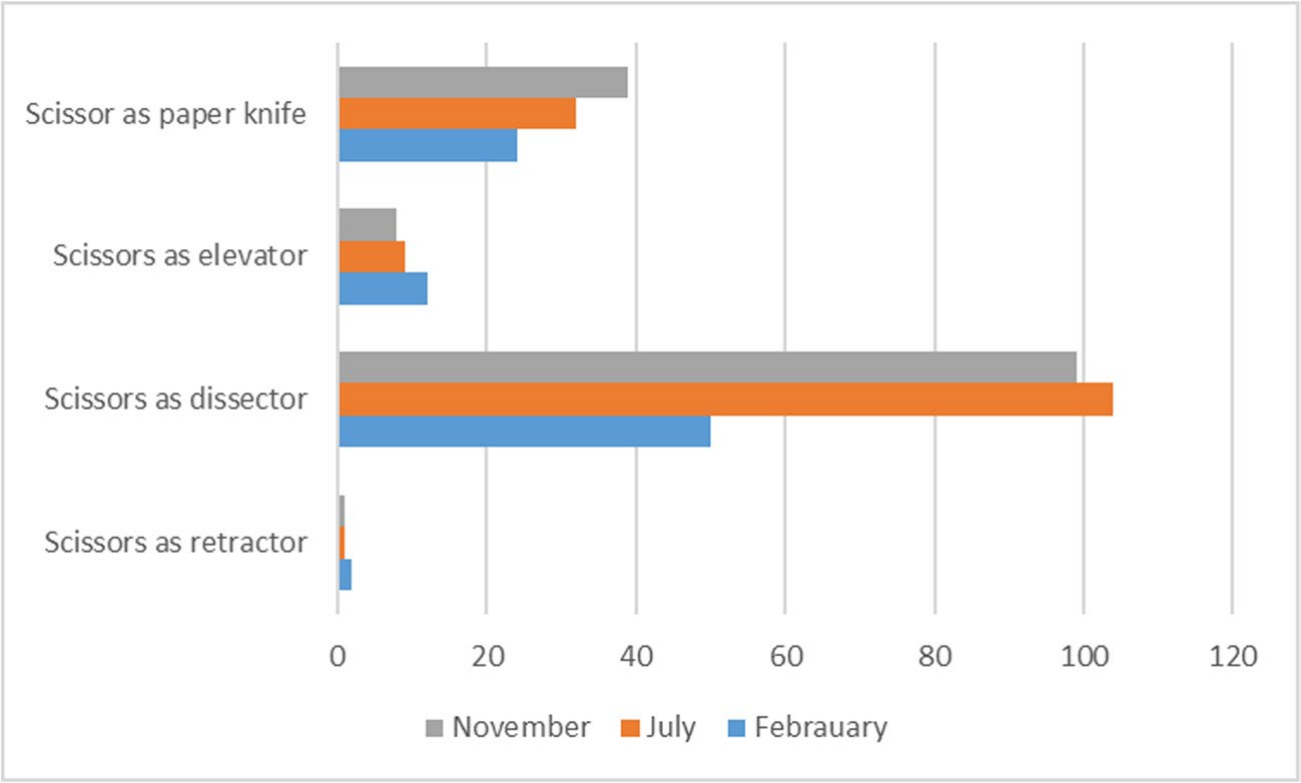
Changes in dissection strategies

We also separately analyzed the metrics that belong to the hand and instrument movements, such as using the scissors as a paper knife, using the scissors as an elevator, using the scissors as a dissector, and using the scissors as a retractor. The changes in dissection strategies are shown in Fig. 5.

The number of unnecessary movements increased in the middle of the training period and decreased towards the end. This was related on the one hand to the increasing familiarity with the exoscope (i.e., less worry about pushing the wrong button); on the other hand, it reflected the progress towards more efficient microsurgical movements which were more pronounced at the end.

In microsurgical movements, we saw several interesting patterns. As the participants learned how to get directly to the target more quickly, the number of repetitive movements and attempts to find the dissection entry zone decreased with experience. With increasing skill and confidence levels, the dissection strategy changed towards a more efficient method, where blunt dissection was substituted by sharp dissection. This reflected also on the versatility of the instrument use. Microscissors were used more actively for multiple tasks at the end of the trial, less frequently for simple cutting but more frequently for dissecting, splitting and stretching. A major change by the end of the trial was the increased use of microscissors for peeling in a paper knife like motion which meant less need for actual cutting, handling of trabecular tissue and using forceps for pulling. The majority of these changes were the result of improvements in micromotor skills, a greater level of trust in the abilities of instruments, and a greater level of confidence in complex movements.

We found that the following variables correlated with the dissection time: going out of focus (*p* = 0.00), unnecessary movements (*p* = 0.002), zooming in (*p* = 0.031), zooming out (*p* = 0.021), repeating movements (*p* = 0.035), searching for entry zone (*p* = 0.00), skeletonizing movements (*p* = 0.00), stretching (*p* = 0.017), splitting (*p* = 0.00), using the microscissors as a paper knife (*p* = 0.011), and cutting (*p* = 0.00).

## Discussion

Digital 3D exoscopes are constantly gaining new advocates from neurosurgeons, especially among the younger generation. To be adopted efficiently, the exoscopic system needs to be tamed and mastered.

It is the first study to evaluate the long-term effects of dedicated practice on microsurgical skills with an exoscope. Our main findings were that with practice the micromotor movements became more efficient, the use of microinstruments became more versatile and the overall work became quicker. Interesting to note, as users gained experience, they manipulated the exoscope’s adjustments more frequently, but the overall pace of the work did not slow down.

It has only been a few years since 3D exoscopes were introduced in clinical neurosurgery. Previous publications have identified strengths of the exoscopic surgery: work ergonomics, teaching, higher magnification, better illumination at depth, wider working angles, and more potential for digital image processing [1, 2, 10, 14,16,4-]. These new intraoperative visualization tools require extensive training and usage to master [1, 2]. Achieving proficiency and excellence requires repetition, creativity, and decision-making skills. However, the operating room is not the place for initial acquisition and refinement of surgical skills using a new surgical system [12, 13, 15]. The safety and predictability of operative procedures for new users of the exoscope necessitate simulation-based training. Dedicated laboratory training is aimed at shifting the early learning curve of a new technology or procedure from the operating room to a venue where rapid repetition is possible and safe.

When planning the test task, we aimed to simulate real microsurgical techniques as much as possible. We wanted a task that challenged the hand-eye coordination, instrument handling, and required active exoscope adjustments. That is why, we selected a dissection model. A dissection technique is a fundamental skill for neurosurgeons. It is used constantly during any microsurgical procedure whether it is vascular, tumor, or spine surgery. Sylvian fissure opening, tumor dissection, and aneurysm clipping are just some classical examples of where a combination of various dissection strategies is involved. Regarding the dissection technique and development of manual skills, we focused on handling the vessels, following the principle of the integrated multi-maneuver dissection technique [3]. Advantages of our model were as follows: easy availability, reproducibility, ease of setup, tissue feel, lack of legislative problems (chicken wings are food), and low cost. With respect to the exoscope use, the experimental task required repetitive camera movement, zoom and focus adjustments, and optimizing working posture like in real surgery. The only downside of the model was that most of the work was done in the same plane without the need for adjusting the camera angle.

The improvements in technique came on the one hand from actual microsurgical skills and on the other hand from better handling of the exoscope. Our test subjects were both novices in the use of exoscopes, but both had years of experience with microsurgery. Our results showed that even neurosurgeons with experience in microsurgery will benefit from dedicated long-term laboratory practice to improve their microsurgical skills further. The most visible trend was the shift from dull dissection into a sharp dissection technique. This is a more demanding technique from a dexterity point of view. In addition, our results also showed that switching from a microscope to an exoscope does not happen immediately but requires time. At the beginning, the users were concerned about making any additional adjustments to the device during the test tasks. As the users gained experience, they utilized more of the device’s features and became less apprehensive about trying new actions.

When analyzing the results of our study, we saw that from the beginning to the end of the experience a 24% reduction in time was obtained with intensive training. However, the question was not just about the time saved by training but also about in which the dissection technique was changed. Different tasks were performed (microvascular anastomosis on chicken wings vessels) with similar results. As a result, we believe that the acquired microsurgical dexterity can also be transferred to other procedures.

We found several trends in how the dissection technique changed over time with practice. Evaluating various aspects of the dissection technique gave us insight into which actions seem to develop and strengthen over time and which actions become more suppressed. These findings can be used to plan more efficient training programs in the future. Simulator training should form an integral part of microsurgical exoscopic surgery training. Simulators have the potential to decrease the learning curve for acquiring exoscopic skills. It can supplement the hands-on training of the clinical phase and act as a bridge between preclinical and clinical training without jeopardizing patients' safety. However, more procedure-specific simulation training is needed in a cost-effective manner.

## Limitations

The dissection task itself has no specific criteria for how to execute it the right way. As a result, we compared the changes over time with no reference criteria other than the final result (harvesting the two arteries without damaging the vessels). We selected the parameters for analysis based on our previous experience of analyzing dissection techniques for Sylvian fissure [3]. Our model did not factor in variables such as hemostasis or some other steps in real surgery. The procedure itself was static without major exoscope camera angular movements. In real surgery, the movement of the camera is essential and affects the workflow significantly. By selecting a relatively static task, we wanted to dilute the effect of the exoscope system used. Each exoscope system has a different way of moving the camera. However, the 3D visualization is very similar across them. Our test task was designed to emphasize how one adjusts to the on-screen 3D image instead of moving the exoscope around.

## Conclusion

Continuous, long-term training is effective for microsurgical skill development when switching from a microscope to an exoscope. With practice, micromotor movements become more efficient and handling microinstruments more versatile. Even users with previous microsurgical experience benefit from dedicated training. Understanding which micromovements develop and strengthen and which become more suppressed with practice helps in designing more efficient microsurgical training tasks.

## Supplementary information

ESM 1

## Data Availability

Raw data of all experiments and statistical analyses are available upon request.

## Abbreviations

SD: Standard deviation
OR: Operation room
3D: Digital three-dimensional
